# Supplementary material for: Transcriptomic profiling of PBDE-exposed HepaRG cells unveils critical lncRNA- PCG pairs involved in intermediary metabolism
Source: PLoS One. 2020 Feb 26;15(2):e0224644. doi: 10.1371/journal.pone.0224644 (PMC7043721; doi:10.1371/journal.pone.0224644)
Supplement: S1 Fig — (PPTX) [file pone.0224644.s001.pptx]

## Slide 1
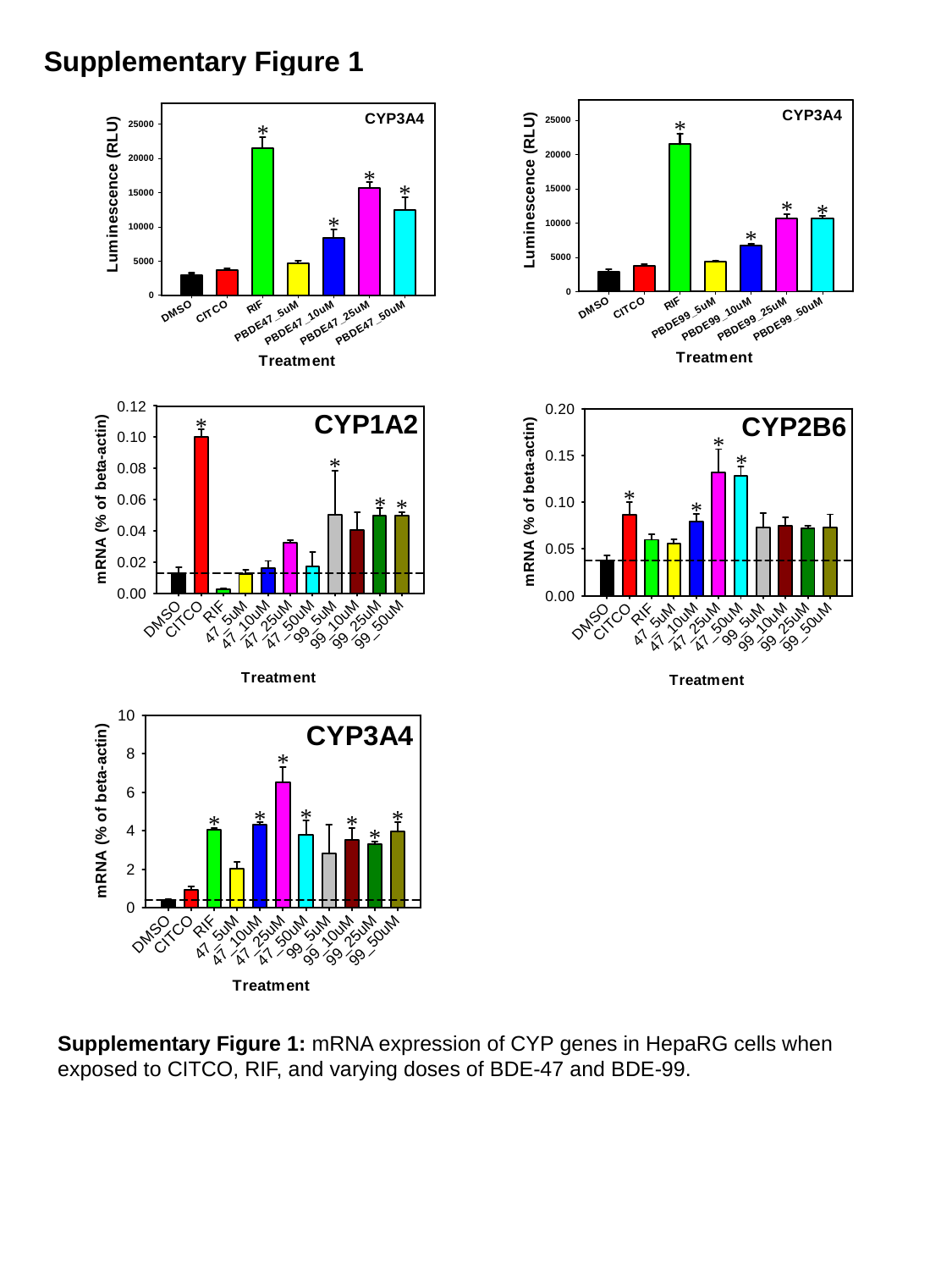

Supplementary Figure 1
Supplementary Figure 1: mRNA expression of CYP genes in HepaRG cells when exposed to CITCO, RIF, and varying doses of BDE-47 and BDE-99.
