# Supplementary material for: Transcriptomic profiling of PBDE-exposed HepaRG cells unveils critical lncRNA- PCG pairs involved in intermediary metabolism
Source: PLoS One. 2020 Feb 26;15(2):e0224644. doi: 10.1371/journal.pone.0224644 (PMC7043721; doi:10.1371/journal.pone.0224644)
Supplement: S2 Fig — (PPTX) [file pone.0224644.s002.pptx]

## Slide 1
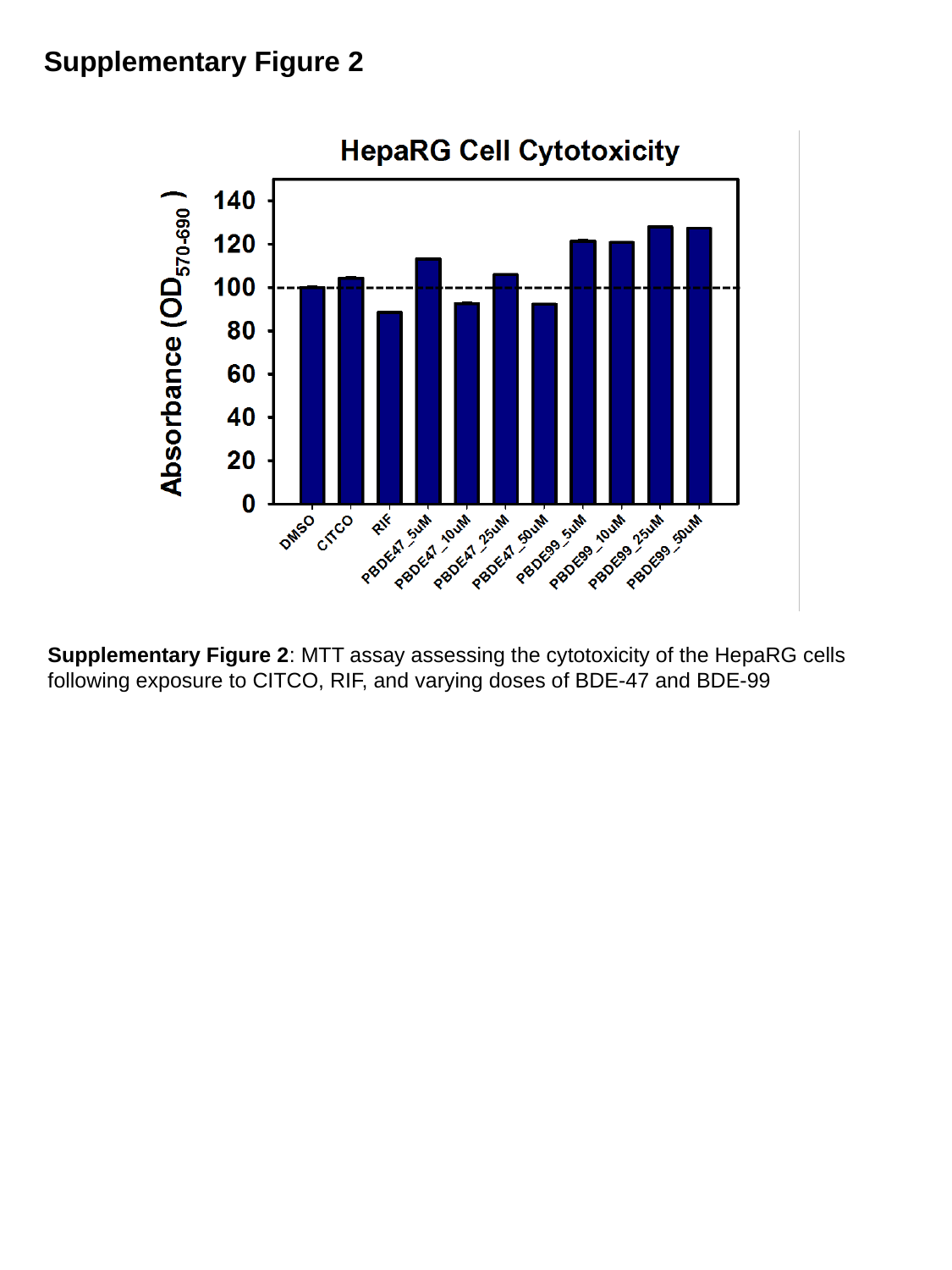

Supplementary Figure 2
Supplementary Figure 2: MTT assay assessing the cytotoxicity of the HepaRG cells following exposure to CITCO, RIF, and varying doses of BDE-47 and BDE-99
